# DP-AMF: Depth-Prior–Guided Adaptive Multi-Modal and Global–Local Fusion for Single-View 3D Reconstruction

**DOI:** 10.3390/jimaging11070246

**Published:** 2025-07-21

**Authors:** Luoxi Zhang, Chun Xie, Itaru Kitahara

**Affiliations:** 1 Doctoral Program in Empowerment Informatics, University of Tsukuba, 1-1-1 Tennodai, Tsukuba 3058577, Japan; 2 Center for Computational Science, University of Tsukuba, 1-1-1 Tennodai, Tsukuba 3058577, Japan

**Keywords:** single-view reconstruction, multi-model, 3D vision, visual feature fusion, indoor scene

## Abstract

Single-view 3D reconstruction remains fundamentally ill-posed, as a single RGB image lacks scale and depth cues, often yielding ambiguous results under occlusion or in texture-poor regions. We propose DP-AMF, a novel Depth-Prior–Guided Adaptive Multi-Modal and Global–Local Fusion framework that integrates high-fidelity depth priors—generated offline by the MARIGOLD diffusion-based estimator and cached to avoid extra training cost—with hierarchical local features from ResNet-32/ResNet-18 and semantic global features from DINO-ViT. A learnable fusion module dynamically adjusts per-channel weights to balance these modalities according to local texture and occlusion, and an implicit signed-distance field decoder reconstructs the final mesh. Extensive experiments on 3D-FRONT and Pix3D demonstrate that DP-AMF reduces Chamfer Distance by 7.64%, increases F-Score by 2.81%, and boosts Normal Consistency by 5.88% compared to strong baselines, while qualitative results show sharper edges and more complete geometry in challenging scenes. DP-AMF achieves these gains without substantially increasing model size or inference time, offering a robust and effective solution for complex single-view reconstruction tasks.

## 1. Introduction

Single-view 3D reconstruction holds significant value in virtual reality, augmented reality, and robotic navigation, especially when multi-view images are unavailable or data acquisition is limited [1,2]. Furthermore, 3D reconstruction is widely needed across various engineering and applied domains, such as building stock estimation [3,4], urban analysis [5], building-integrated photovoltaics (BIPV) design [6], solar potential analysis [7,8], precision agriculture [9] and biomedical imaging [10].

However, inferring 3D geometry from a single RGB image is inherently ill-posed; the image lacks direct depth information, and in cluttered or heavily occluded environments, 2D features are often corrupted by noise, making it difficult to recover hidden regions and fine-grained structures accurately.

Existing methods can be broadly categorized into two approaches: (1) relying solely on CNNs or Vision Transformers (ViTs) to extract RGB features before reconstructing 3D structure via implicit or explicit decoders; (2) first using a monocular depth estimator (e.g., MiDaS [11]) to generate a depth map, and then performing joint reconstruction. Pure RGB-based approaches often produce incomplete geometry or lose fine details in texture-deficient or occluded regions [12]. Meanwhile, traditional monocular depth networks generate depth maps with limited global consistency and poor edge sharpness, which can exacerbate reconstruction errors. Despite recent advances, two critical challenges remain largely unresolved: (i) existing depth priors (e.g., MiDaS, DPT) are limited by noisy boundaries and poor global coherence, hindering precise geometry estimation; (ii) fixed fusion methods, such as simple concatenation, cannot effectively adapt the importance of RGB, depth, and global semantic features according to local texture and occlusion conditions, leading to suboptimal performance in complex scenarios. Clearly addressing these gaps is essential to improving the robustness and quality of single-view reconstruction.

In addition, realistic surface appearance is crucial for downstream applications. Oechsle et al. [13] propose Texture Fields, a continuous 3D function representation that decouples texture from mesh discretization and regresses per-point color values, enabling high-frequency detail reconstruction in implicit models. This line of work suggests future extensions where geometry and texture are inferred jointly in a unified framework.

To further improve single-view reconstruction accuracy, it is necessary to introduce higher-fidelity depth priors and achieve a dynamic balance between global and local information during feature fusion.

To this end, we propose the DP-AMF framework, Depth-Prior-Guided Adaptive Multi-Modal and Global–Local Fusion. First, we employ the publicly released pre-trained weights of the MARIGOLD diffusion-based depth estimator to generate high-fidelity depth maps [14]. These depth priors provide reliable spatial cues without extra training overhead. Next, we design an adaptive fusion encoder that concatenates local features extracted by ResNet, global features from DINO-ViT, and the depth priors. A learnable channel-wise weighting module automatically determines which source to rely on at each location: emphasizing RGB details in texture-rich areas and depending more on depth priors and global context in texture-poor or heavily occluded regions. The fused features are then passed to an implicit signed distance field (SDF) decoder to generate the final mesh. While DP-AMF focuses on enhancing geometric fidelity, future integration of methods like Texture Fields [13] could further endow reconstructed meshes with high-quality textures.

In fair comparisons against other implicit reconstruction baselines, DP-AMF demonstrates superior performance on the 3D-FRONT and Pix3D datasets; Chamfer Distance (CD) is reduced by 3.5%, F-Score increases by 2.6%, and Normal Consistency (NC) improves by 0.9% [15,16,17]. Qualitative results further show sharper edges and more complete geometry under heavy occlusion. DP-AMF thus significantly enhances single-view reconstruction accuracy and detail fidelity.

The main contributions of this work are:

- **Depth-Prior Multi-Modal Fusion:** We use the pre-trained MARIGOLD diffusion-based depth estimator to generate high-fidelity depth priors [14], which are concatenated with RGB features to alleviate the ill-posed nature of single-view reconstruction.
- **Adaptive Global–Local Feature Fusion:** Our encoder processes ResNet-based local features and DINO-ViT global features in parallel, then merges them with pixel-wise depth priors via a learnable fusion module that dynamically adjusts information weights according to texture and occlusion.
- **Significant Performance Improvements:** We validate the effectiveness of our approach in fair comparisons with other implicit reconstruction baselines, demonstrating that DP-AMF outperforms existing methods on key metrics (CD, F-Score, NC) [15,16,17] and achieves higher reconstruction quality and detail fidelity in complex scenes.

The rest of this paper is organized as follows. Section 2 reviews prior work on single-view 3D reconstruction and related texture modeling. Section 3 introduces our DP-AMF framework, including depth prior generation, feature extraction, and the adaptive fusion module. Section 4 describes the evaluation protocol; in Section 4.1 we detail the 3D-FRONT and Pix3D datasets, and in Section 4.2 we specify implementation and training settings. Section 5 presents results and analysis; in Section 5.1 we compare against state-of-the-art baselines, and in Section 5.2 we conduct ablation studies to isolate each component’s effect. Finally, Section 6 discusses limitations, potential applications, and future work.

## 2. Related Work

### 2.1. Multi-Modal Single-View Reconstruction

Early single-view 3D reconstruction methods relied solely on RGB features, mapping images to volumetric or point-based representations via CNNs or Vision Transformers (ViTs), such as Pix2Vox [18], IV-Net [19], and AtlasNet [20]. However, in texture-sparse or heavily occluded scenarios, these RGB-only approaches often produce incomplete geometry or lose fine details [12,21]. Subsequent work introduced monocular depth priors (e.g., MiDaS [11], DPT [22,23]) to compensate for missing depth information. For example, Kim et al. [24] used gradients from MiDaS depth maps to enhance local reconstruction accuracy. Yet these methods frequently suffer from limited global consistency in the depth maps or over-reliance on depth, which can suppress RGB-driven texture details.

In contrast, we adopt the pre-trained MARIGOLD diffusion-based depth estimator [14] to generate high-fidelity depth maps. Diffusion priors provide stronger robustness and detail fidelity than MiDaS or other monocular depth networks. Within our network, each depth map is encoded into a high-dimensional feature and then propagated to 3D sample points via linear interpolation, supplying reliable geometric constraints at every point without diminishing RGB-driven texture expressions.

### 2.2. Global–Local Feature Extraction

In single-view reconstruction, pure CNNs (e.g., ResNet [25]) excel at capturing local texture but struggle to encode global shape priors, while pure ViTs [26] model global context effectively but often lack fine edge details. To address this trade-off, prior works have fused both. Yang et al. [27] concatenated ResNet and ViT features before feeding them into an implicit decoder. However, fixed concatenation or simple addition fails to adaptively balance local and global information in texture-rich versus occluded regions.

Our DP-AMF encoder proceeds in three stages. First, it extracts shallow features via a CNN; next, these features are fed in parallel to DINO-ViT [28] for global semantics and to ResNet for deeper local details; finally, the three streams—global, local, and depth priors—are fused by a learnable channel-wise weighting module that dynamically allocates importance based on texture density and occlusion. Ablation studies demonstrate that this adaptive fusion outperforms fixed concatenation or simple addition in preserving both global contours and local details.

## 3. Methodology

**Figure 1:**
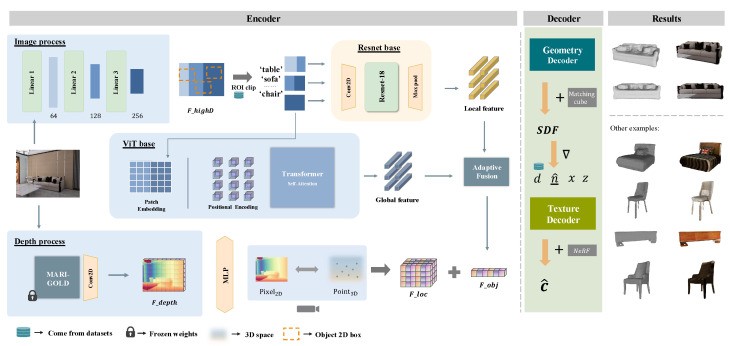
Overall pipeline of the proposed DP-AMF framework. From left to right: (1) MARIGOLD depth-prior branch; (2) shallow CNN, DINO-ViT and ResNet-18 branches; (3) adaptive global–local fusion; (4) two-stage implicit reconstruction for geometry and texture.

Figure 1 depicts the overall DP-AMF pipeline. We address two main challenges in single-view 3D reconstruction: (1) the lack of reliable depth cues from a single RGB image, and (2) the need to balance fine local details with global context. Accordingly, we introduce (i) a depth-prior branch using a diffusion-based estimator to supply high-fidelity geometric guidance, and (ii) an adaptive fusion encoder that integrates local, global, and depth features in a data-driven manner. In this section, we explain our design choices and their motivations step by step.

### 3.1. Depth Prior Generation

Single-view reconstruction is ill-posed because an RGB image alone lacks scale and depth information, especially in texture-poor or occluded regions. Prior works used MiDaS or DPT to generate depth maps, but those networks often yield noisy edges and inconsistent global structure under challenging conditions. To obtain a more reliable depth prior, we adopt the MARIGOLD diffusion-based depth estimator [14]. Diffusion priors have shown superior robustness to occlusions and lighting variations compared to earlier monocular depth methods.

Concretely, given an input image *x*, MARIGOLD first encodes *x* into a latent z(x) via a VAE encoder. We initialize the depth latent $z{\left(d\right)}_{T}$ with Gaussian noise and iteratively denoise it conditioned on z(x):

(1) $$z{\left(d\right)}_{t-1}=G_{\theta }\left(z{\left(d\right)}_{t},\;t,\;z\left(x\right)\right),\;t=T,T-1,\ldots ,1,$$

where $G_{\theta }$ is the learned denoiser. After *T* steps, the final latent $z{\left(d\right)}_{0}$ is decoded into a depth map:

(2) $$\hat{d}=D\left(z{\left(d\right)}_{0}\right),$$

with *D* the VAE decoder. We then apply a 3×3 convolution to convert $\hat{d}$ (size H×W) into a multi-channel depth feature $F_{\mathrm{depth}}\in R^{H^{\prime }\times W^{\prime }\times C_{d}}$. This convolution both increases the representational capacity (from 1 channel to $C_{d}$ channels) and downsamples to match the resolution $H^{\prime }\times W^{\prime }$ of later feature maps.

To reduce training and inference overhead, all MARIGOLD depth maps are computed once offline using the pre-trained model and cached; they are neither re-generated nor fine-tuned during subsequent training or inference.

By freezing $F_{\mathrm{depth}}$, i.e., not fine-tuning MARIGOLD, we ensure stable, high-quality depth guidance without extra training cost. In practice, when sampling a 3D point $p_{i}$ along the camera ray,

(3) $$p_{i}=o+t_{i}\;d,\;t_{i}∼\mathrm{Uniform}\left(t_{\min},t_{\max}\right),$$

we project $p_{i}$ onto the image plane using $\pi (p_{i})$ and fetch the corresponding depth feature by bilinear interpolation:

(4) $$F_{\mathrm{depth}}\left(p_{i}\right)\;=\;\mathrm{Bilinear}\left(F_{\mathrm{depth}},\;\pi \left(p_{i}\right)\right).$$

This assignment gives each 3D sample point a robust geometric cue that significantly improves geometry estimation under occlusion or low texture.

**Figure 2:**
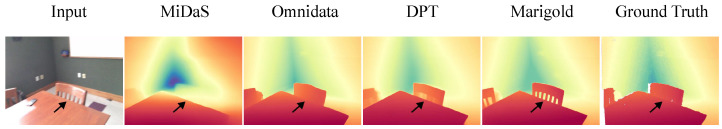
Qualitative comparison of monocular depth priors on a representative indoor scene. Arrows highlight regions where MARIGOLD more faithfully preserves object boundaries and reduces noise in occluded areas, demonstrating its superior object-level detail and global consistency [14].

We select MARIGOLD as our depth-prior generator for three primary reasons. (i) Compared to conventional estimators such as MiDaS or DPT, MARIGOLD’s diffusion-based denoising mechanism exhibits superior robustness to occlusions and challenging lighting, yielding more accurate object-level depth details; (ii) being derived from Stable Diffusion, MARIGOLD retains rich visual priors that enable strong zero-shot generalization across diverse, unseen scenes; (iii) to minimize computational overhead, we precompute and cache all MARIGOLD depth maps offline—using even the lightest publicly available pre-trained variant requires only 0.87 s per image—thereby achieving a favorable balance between accuracy and efficiency. Figure 2 presents a side-by-side qualitative comparison of depth maps produced by MiDaS, Omnidata, DPT, and MARIGOLD against ground truth. As highlighted by the arrows, MARIGOLD more faithfully delineates object edges (e.g., chair back) and reduces background noise, validating its selection as the most effective depth prior in our framework.

### 3.2. Feature Extraction and Fusion

Reconstructing fine geometry requires features that capture both high-frequency local detail and low-frequency global context. We therefore design a two-step encoder; first extract local and global features separately, then merge them adaptively with depth.

**Local vs. Global Extraction Rationale.** Early CNNs (e.g., VGG or ResNet) excel at capturing local texture and edges, making them ideal for fine-grained geometry. However, they lack a mechanism to model long-range dependencies, which can cause shape inference inconsistencies in large, complex scenes. Vision Transformers (ViTs) address this by computing global self-attention, but a standard ViT pre-trained on classification (e.g., ImageNet) may not robustly encode precise boundary details. We choose DINO-ViT [28]—a self-distilled ViT variant—because its unsupervised training learns more semantically consistent patch representations, improving robustness under occlusion and lighting changes.

**Shallow Feature Backbone.** We begin by extracting shallow features with ResNet-32 up to layer *k*:

(5) $$F_{\mathrm{shallow}}=\mathrm{ResNet}32_{\mathrm{layers}1\;-\;k}\left(x\right),$$

where $F_{\mathrm{shallow}}\in R^{H^{\prime }\times W^{\prime }\times C_{s}}$. We use ResNet-32 instead of a deeper network because we only need to capture mid-level textures; deeper layers would aggregate too much semantic abstraction and discard edges essential for geometry.

**Global Feature via DINO-ViT.** To capture scene-wide context, we pass $F_{\mathrm{shallow}}$ through DINO-ViT. Specifically, we flatten or patchify $F_{\mathrm{shallow}}$ to form ViT inputs, obtain the learned global CLS token $C_{\mathrm{vit}}\in R^{D}$, and project it back to spatial dimensions with a 1×1 convolution:

(6) $$F_{\mathrm{vit}}=\mathrm{Conv}1\times 1\left(C_{\mathrm{vit}}\right),\;F_{\mathrm{vit}}\in R^{H^{\prime }\times W^{\prime }\times C_{v}},$$

This $F_{\mathrm{vit}}$ encodes long-range dependencies, enabling coherent shape reasoning across the entire image. **Local Feature via ResNet-18.** In parallel, $F_{\mathrm{shallow}}$ is fed into ResNet-18 from layer 1 to *m* to extract deeper local features:

(7) $$F_{\mathrm{res}}=\mathrm{ResNet}18_{\mathrm{layers}1\;-\;m}\left(F_{\mathrm{shallow}}\right),\;F_{\mathrm{res}}\in R^{H^{\prime }\times W^{\prime }\times C_{r}}.$$

We choose ResNet-18 for local detail because its residual connections help preserve edge information, and it is lightweight enough to avoid overfitting on small datasets.

As shown in Figure 1, our encoder employs a two-stage feature extraction strategy. First, ResNet-32 processes the entire image to produce shallow global features $F_{\mathrm{highD}}$ that capture scene layout and identify regions of interest (e.g., tables, sofas, chairs). Then, each ROI is further refined by ResNet-18 to generate high-fidelity, 256-dimensional local object features $F_{\mathrm{obj}}$. This hierarchical design leverages ResNet-32’s capacity for broad contextual reasoning alongside ResNet-18’s strength in preserving fine-grained details, yielding a compact yet expressive representation without the parameter overhead of a single deeper network or the loss of context from using only ResNet-18.

**Adaptive Fusion of Three Modalities.** Having obtained the three feature maps $\left\{F_{\mathrm{vit}},F_{\mathrm{res}},F_{\mathrm{depth}}\right\}$ with channels $\left(C_{v},C_{r},C_{d}\right)$, we concatenate them:

(8) $$F_{\mathrm{cat}}=\left[F_{\mathrm{vit}}\;∥\;F_{\mathrm{res}}\;∥\;F_{\mathrm{depth}}\right]\;\in \;R^{H^{\prime }\times W^{\prime }\times \left(C_{v}+C_{r}+C_{d}\right)}.$$

A 1×1 convolution followed by Sigmoid produces channel-wise weights

(9) $$\alpha =\sigma \left(\mathrm{Conv}1\times 1(F_{\mathrm{cat}})\right),\;\alpha \in {\left[0,1\right]}^{C_{v}+C_{r}+C_{d}},$$

where $\alpha_{c}$ indicates the relative importance of channel *c*. The fused feature is

(10) $$F_{\mathrm{fusion}}=\sum_{c=1}^{C_{v}+C_{r}+C_{d}}\alpha_{c}\;F_{\mathrm{cat}}^{\left(c\right)},\;F_{\mathrm{fusion}}\in R^{H^{\prime }\times W^{\prime }\times C_{f}}.$$

**Figure 3:**
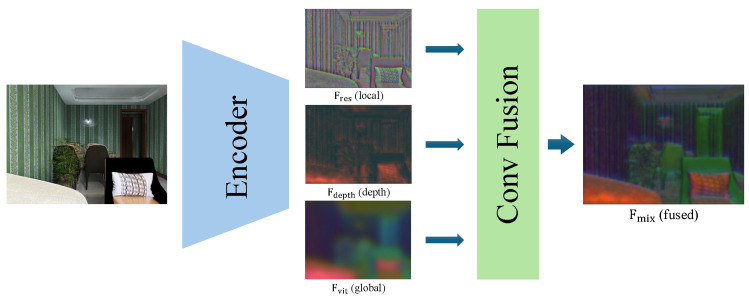
Visualization of our adaptive fusion process. From left to right: the global-vision feature $F_{\mathrm{vit}}$, the local-detail feature $F_{\mathrm{res}}$, the depth-sensitive feature $F_{\mathrm{depth}}$, and the final fused feature $F_{\mathrm{mix}}$ after the 1×1 convolution.

This adaptive weighting ensures that in texture-rich regions (where local detail matters), $F_{\mathrm{res}}$ channels receive higher weights, while in occluded or uniform areas, $F_{\mathrm{vit}}$ or $F_{\mathrm{depth}}$ channels dominate. Compared to fixed concatenation (which treats all channels equally), this mechanism dynamically balances the three modalities based on the local context. Figure 3 shows a qualitative illustration of the three input modalities and their fusion.

Although recent works explore complex cross-modal fusion (e.g., transformer-based attention), we adopt a simple concatenation followed by a 1×1 convolution and sigmoid activation for channel-wise weighting. This choice is motivated by the spatially aligned and complementary nature of our ResNet, ViT, and depth features, making a lightweight fusion both effective and efficient. To prevent the learned weights from collapsing to uniform or single-modality distributions, we clamp the pre-sigmoid logits to [−3,3] and apply an L2 weight decay of $1\times 10^{-4}$ on the fusion layer parameters. These measures ensure sufficient diversity in the per-channel weights, allowing the network to adaptively emphasize the most informative modality at each spatial location.

### 3.3. Two-Stage Training Objectives

We train DP-AMF in two stages to decouple geometry learning from appearance and avoid geometry-texture coupling.

**Stage 1: Geometry-Only Optimization.** We represent geometry with a signed distance function (SDF) network $h_{\theta }$, which takes as input a 3D point p and its fused feature:

$$s\left(p\right)=h_{\theta }\left(p,\;F_{\mathrm{fusion}}\left(\pi \left(p\right)\right)\right),$$

where π(p) projects p to image coordinates and fetches $F_{\mathrm{fusion}}$. We minimize the L1 SDF loss:

(11) $$L_{\mathrm{sdf}}=E_{p∼P}\left|s\left(p\right)-s^{*}\left(p\right)\right|,$$

where $s^{*}\left(p\right)$ is the ground-truth signed distance. By focusing solely on geometry in Stage 1, we prevent early texture gradients from distorting the shape.

**Stage 2: Full Reconstruction.** Once geometry training loss converges to a stable minimum (monitored empirically), we fix $h_{\theta }$ momentarily and introduce three additional losses: color, normal consistency, and depth consistency. The total loss is

(12) $$L=\lambda_{\mathrm{sdf}}\;L_{\mathrm{sdf}}+\lambda_{\mathrm{color}}\;L_{\mathrm{color}}+\lambda_{\mathrm{normal}}\;L_{\mathrm{normal}}+\lambda_{\mathrm{depth}}\;L_{\mathrm{depth}}.$$

Here, $L_{\mathrm{color}}$ is the photometric error between the rendered color and ground truth. $L_{\mathrm{normal}}$ measures the angular difference between predicted normals ∇s(p) and ground-truth normals. $L_{\mathrm{depth}}$ enforces consistency between the 2D-projected depth from the implicit SDF and the diffusion-based depth $\hat{d}$.

Specifically, on the large-scale synthetic 3D-FRONT dataset, geometry typically converges at approximately epoch 30. Thus, Stage 2 begins at epoch 30, gradually ramping up appearance-related losses until reaching their full weights by epoch 80, and continuing training through epoch 200. For the smaller yet more complex Pix3D dataset, geometry convergence occurs later—around epoch 50; accordingly, appearance losses are introduced at epoch 50, fully weighted by epoch 150, and training continues until epoch 300 (see Table 1).

Preliminary experiments indicated that freezing the geometry network before introducing appearance losses maintains stable geometric fidelity. Attempting end-to-end fine-tuning (i.e., allowing appearance gradients into geometry from the outset) led to slower convergence of geometry and provided no substantial improvements in final reconstruction quality. We attribute this to conflicting gradients from geometric and appearance objectives during early training stages.

Although our staged training approach improves stability and ensures clear geometry–texture decoupling, we acknowledge that joint optimization could potentially allow for greater holistic feature sharing. Exploring this balance further constitutes an interesting avenue for future research.

## 4. Experiments

### 4.1. Datasets

We evaluate DP-AMF on two widely used benchmarks covering both synthetic and real indoor scenes: **3D-FRONT** [29] and **Pix3D** [30].

**3D-FRONT** contains over 10,000 synthetic indoor scene models and more than 300,000 individual 3D objects across diverse categories with detailed material and layout annotations. It also provides precise camera poses and both 2D/3D bounding boxes, which facilitate reliable spatial relationship learning and occlusion handling.

**Pix3D** offers 12,471 real-world image–model pairs spanning 9 object categories (e.g., chairs, tables, sofas) with fine-grained pixel-to-model alignments. We adopt the standard split of 6931 (55.6%) training, 2778 (22.3%) validation, and 2762 (22.1%) testing samples [30].

Following the split scheme of Liu et al. [31], we randomly split these datasets as shown in Table 2.

### 4.2. Experimental Setup

All experiments were run under Ubuntu 20.04 on an NVIDIA RTX 3090 GPU. We leverage PyTorch (version 2.5.1, developed by Facebook AI Research, Menlo Park, CA, USA) with CUDA and multi-threaded data loading for efficiency. For full reproducibility, we provide detailed training and implementation configurations in Table 3. We also measured the wall-clock inference time on the RTX 3090; over 2000 runs on the 3D-FRONT dataset, geometry combined with texture inference takes around 1.647 s per image.

## 5. Results and Analysis

### 5.1. Compared Experiments

We evaluate our method against four state-of-the-art single-view 3D reconstruction approaches—MGN [32], LIEN [33], InstPIFu [31], and SSR [12]—under identical training and testing configurations on both the 3D-FRONT and Pix3D datasets. These baselines were chosen because they all target indoor scene data, employ implicit surface representations, and emphasize either holistic scene understanding or high-fidelity object reconstruction.

While more recent implicit reconstruction methods such as POCO [34] and Neural Kernel Surface Reconstruction (NKSR) [35] have been proposed, these techniques primarily operate on 3D point-cloud inputs, focusing explicitly on tasks involving sparse and noisy 3D measurements or large-scale point-cloud data. In contrast, our method and the selected baselines (MGN, LIEN, InstPIFu, SSR) specifically target single-view RGB image inputs, optionally enhanced by depth priors. Directly comparing with POCO or NKSR would thus involve fundamentally different input modalities, task definitions, and experimental setups, potentially resulting in confounding factors.

As summarized in Table 4 and Table 5, we compare CD, F-Score, and NC. In both datasets, our method achieves the lowest CD, highest F-Score, and superior NC. The improvements—highlighted in bold in the tables—demonstrate the effectiveness of our global–local feature fusion and depth-guided alignment over prior work.

**Figure 4:**
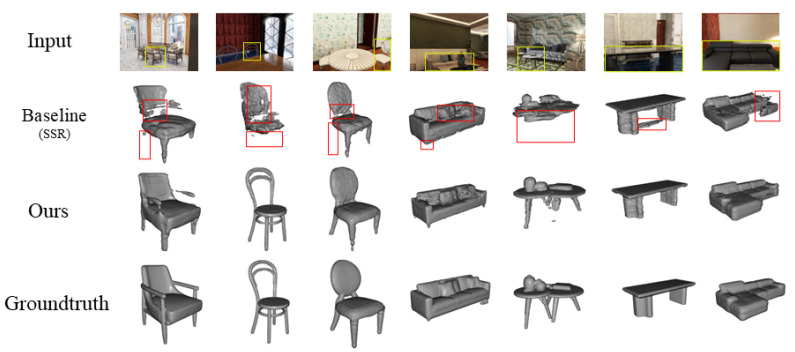
Reconstruction results of indoor objects on the 3D-FRONT dataset [29]. We compare our method against the strong baseline SSR [12]. Red bounding boxes highlight regions with notable differences, illustrating our improved recovery of fine geometric details.

**Figure 5:**
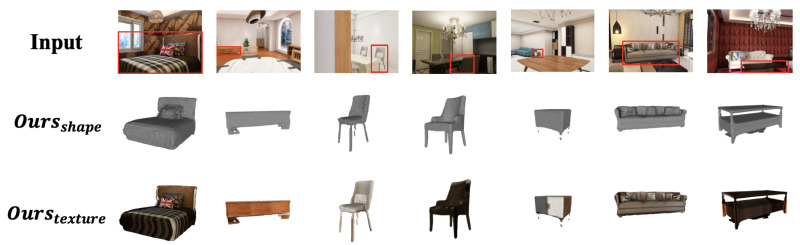
In the second stage of indoor object reconstruction, the proposed model generates high-quality textured 3D objects. Red bounding boxes highlight the regions of our reconstructed objects.

For qualitative comparison, we select SSR as the visualization baseline due to its strong performance and representative architecture. Figure 4 shows reconstructed scenes from SSR (the second row) versus our method (the third row). Notably, our fusion of global context and local detail yields more complete object geometry (e.g., the table in the third column from the right) while preserving fine-grained structures (e.g., the sofa in the fourth column from the left). Moreover, incorporating depth information clearly reduces background artifacts—see the chair in the second column from the left, where our method better suppresses background interference. We also showcase additional reconstruction results (geometry and texture) of our method in Figure 5.

For a more detailed analysis, we further compare the additional model complexity introduced by DP-AMF over the strongest baseline SSR. As shown in Table 6, DP-AMF requires only 0.19 M more learnable parameters and incurs an extra 68.41 GFLOPs per forward pass. Despite this modest increase in both parameter count and computational cost, our method consistently outperforms SSR—achieving lower Chamfer Distance and higher F-Score and Normal Consistency—thereby demonstrating that the improvements arise from more effective global–local feature fusion and depth-guided alignment rather than mere scaling of model size.

Overall, our baseline selection provides a fair, focused, and meaningful evaluation of DP-AMF against directly comparable methods in terms of methodological similarities, input modalities, and computational requirements.

### 5.2. Ablation Experiments

We conduct a set of ablation studies on the 3D-FRONT validation split, training each variant for 80 epochs. We examine:

- **Depth backbone**: MARIGOLD [14] vs. MiDaS [11] vs. Without depth module.
- **Global encoder**: ViT [26] vs. DINO-ViT [28] vs. Without global encoder.

Table 7 reports the resulting CD, F-Score, and NC. Adding depth yields a substantial CD reduction and boosts both F-Score and NC. Furthermore, replacing UNICORN with MiDaS provides an additional gain, demonstrating the importance of accurate depth priors. Finally, substituting traditional ViT with DINO-ViT for global encoding further improves all metrics, confirming that self-supervised features better complement local and depth cues.

These ablations confirm that each component of our framework—depth guidance, choice of depth model, and choice of global encoder—contributes positively and synergistically to single-view 3D reconstruction performance.

## 6. Discussion

Our results show that integrating high-fidelity diffusion-based depth priors with an adaptive global–local fusion encoder substantially closes the gap left by RGB-only and fixed-fusion methods. In comparison to prior approaches (e.g., SSR [12], InstPIFu [31]), DP-AMF reduces CD by approximately 7.64%, boosts F-Score by about 2.81%, and improves NC by around 5.88% on the 3D-FRONT dataset. These gains confirm that diffusion priors provide reliable geometric cues under occlusion and that our channel-wise weighting mechanism effectively balances fine textures and global context, enabling more complete and accurate reconstructions in cluttered indoor scenes.

Unlike traditional point cloud-based reconstructions often used in architectural heritage documentation [36], the integration of high-fidelity texture reconstruction can enable more realistic digital preservation [37] and immersive visualization in VR platforms [38]. In urban navigation, where 2D maps or images can be confusing [39], textured 3D models could provide more intuitive spatial orientation and landmark recognition [40]. Furthermore, in fields like structural health monitoring [41], detailed textured meshes can enhance the visibility and tracking of surface-level damages such as cracks or erosion over time [42].

Although we did not include explicit statistical significance tests in this study, we conducted extensive ablation experiments demonstrating that removing any key module consistently degrades CD, F-Score, and NC, confirming the robustness of each component’s contribution. In future work, we plan to perform paired *t*-tests and bootstrap analyses on these metrics to quantitatively evaluate the statistical significance of the observed improvements.

Despite these advances, DP-AMF still depends heavily on large, fully annotated 3D datasets, which limits its out-of-domain generalization. Moreover, the multi-branch encoder and diffusion-based depth generation incur nontrivial computational overhead. Future work could explore semi- or weakly supervised learning methods to reduce annotation demands and investigate lightweight fusion modules or attention distillation techniques to accelerate inference. Extending the framework to dynamic scenes, outdoor environments, or integrating multi-modal cues (e.g., incorporating language–image models such as CLIP [43]) would further broaden its applicability, facilitating real-time augmented reality (AR), VR, and robotic perception tasks.

## Figures and Tables

**Table 1:** Loss configurations and training milestones for the two-stage training scheme.

	$L_{\mathrm{sdf}}$	$L_{\mathrm{color}}$	$L_{\mathrm{normal}}$	$L_{\mathrm{depth}}$
Stage 1	1.0	0	0	0
Stage 2 (3D-FRONT)	1.0	0→0.1 (epochs 30–80)	0→0.01 (epochs 30–80)	0→0.1 (epochs 30–80)
Stage 2 (Pix3D)	1.0	0→0.1 (epochs 50–150)	0→0.01 (epochs 50–150)	0→0.1 (epochs 50–150)

**Table 2:** Dataset splits for 3D-FRONT and Pix3D.

Dataset	Train	Validation	Test
3D-FRONT	22,103 (74.5%)	2550 (8.6%)	5006 (16.8%)
Pix3D	6931 (55.6%)	2778 (22.3%)	2762 (22.1%)

**Table 3:** Experimental setup and hyperparameters.

Item	Configuration
System	Ubuntu 20.04, NVIDIA RTX 3090
Framework	PyTorch + CUDA
Training Stages	2 (Stage 1: Geometry only; Stage 2: Full reconstruction)
Epochs	200(3D-FRONT) 300(Pix3D)
Optimizer	Adam, LR = $1\times 10^{-4}$
Batch Size	96 (3D-FRONT) 128(Pix3D)
Image Resolution	484×648
CNN Backbones	ResNet-32, ResNet-18 (ImageNet pre-trained)
ViT Backbone	DINO-ViT-16 (768-dim CLS)
Depth Prior	MARIGOLD diffusion pretrained model
Point Sampling	N = 64 samples/ray

**Table 4:** Evaluation of object reconstruction on the 3D-FRONT dataset [29]. **Bold** indicates the best performance, underlined the second best, and the shaded row highlights our method.

Metrics	Category	Bed	Chair	Sofa	Table	Desk	Nightstand	Cabinet	Bookshelf	Mean
CD ↓ (7.64%)	MGN	15.48	11.67	8.72	20.90	**17.59**	17.11	13.13	10.21	14.07
	LIEN	16.81	41.40	9.51	35.65	26.63	16.78	11.70	11.70	28.52
	InstPIFu	18.17	14.06	7.66	23.25	33.33	11.73	6.04	8.03	14.46
	SSR	13.12	12.05	6.47	19.32	28.45	11.87	6.18	7.23	13.08
	Ours	**12.05**	**10.89**	**5.94**	**18.47**	26.87	**10.15**	**5.27**	**5.82**	**12.08**
F-Score ↑ (2.81%)	MGN	46.81	57.49	64.61	49.80	46.82	47.91	54.18	54.55	55.64
	LIEN	44.28	31.61	61.40	43.22	37.04	50.76	69.21	55.33	45.63
	InstPIFu	47.85	59.08	67.60	56.43	48.49	57.14	73.32	66.13	61.32
	SSR	52.13	62.47	69.21	60.34	52.78	60.12	75.45	68.09	62.25
	Ours	**54.21**	**64.37**	**71.08**	**62.01**	**54.66**	**62.45**	**76.12**	**69.30**	**64.00**
NC ↑ (5.88%)	MGN	0.829	0.758	0.819	0.785	0.711	0.833	0.802	0.719	0.787
	LIEN	0.822	0.793	0.803	0.755	0.701	0.814	0.801	0.747	0.786
	InstPIFu	0.799	0.782	0.846	0.804	0.708	0.844	0.841	0.790	0.810
	SSR	0.832	0.803	0.849	0.814	0.709	0.861	0.828	0.806	0.813
	Ours	**0.834**	**0.812**	**0.861**	**0.822**	**0.729**	**0.869**	**0.848**	**0.815**	**0.824**

**Table 5:** Evaluation of object reconstruction on the Pix3D dataset [30]. Bold indicates the best performance, underlined the second best, and the shaded row highlights our method.

Metrics	Models	Bed	Bookcase	Chair	Desk	Sofa	Table	Tool	Wardrobe	Misc	Mean
CD ↓ (4.84%)	MGN	22.91	33.61	56.47	33.95	9.27	81.19	94.70	10.43	137.50	44.32
	LIEN	11.18	29.61	40.01	65.36	10.54	146.13	29.63	4.88	144.06	51.31
	InstPIFu	10.90	7.55	32.44	22.09	8.13	45.82	10.29	**1.29**	47.31	24.65
	SSR	6.31	7.21	26.23	28.63	5.68	43.87	8.29	2.07	35.03	21.79
	**Ours**	**6.05**	**6.92**	**25.51**	**27.73**	**5.52**	**42.10**	**7.98**	1.93	**34.12**	**20.83**
F-Score ↑ (2.74%)	MGN	34.69	28.42	35.67	**65.36**	51.15	17.05	57.16	52.04	10.41	36.20
	LIEN	37.13	15.51	25.70	26.01	49.71	21.16	5.85	59.46	11.04	31.45
	InstPIFu	54.99	62.26	35.30	47.30	56.54	37.51	64.24	**94.62**	27.03	45.62
	SSR	68.78	66.69	55.18	42.49	71.22	51.93	65.38	91.84	46.92	59.71
	**Ours**	**69.45**	**67.12**	**56.23**	43.78	**72.04**	**53.87**	**66.05**	92.30	**48.31**	**61.35**
NC ↑ (5.85%)	MGN	0.737	0.592	0.525	0.633	0.756	0.794	0.531	0.809	0.563	0.659
	LIEN	0.706	0.514	0.591	0.581	0.775	0.619	0.506	0.844	0.481	0.646
	InstPIFu	0.782	0.646	0.547	0.758	0.753	0.796	0.639	0.951	0.580	0.683
	SSR	0.825	0.689	0.693	0.776	0.866	0.835	0.645	0.960	0.599	0.778
	**Ours**	**0.831**	**0.696**	**0.702**	**0.781**	**0.871**	**0.842**	**0.652**	**0.965**	**0.610**	**0.791**

**Table 6:** Comparison of parameter count and computational cost between SSR and DP-AMF.

Model	Params (M)	GFLOPs (G)	ΔParams (M)	ΔGFLOPs (G)
SSR	36.29	147.44	–	–
DP-AMF	36.48	215.85	+0.19	+68.41

**Table 7:** Ablation study results on 3D-FRONT after 80 training epochs.

Depth Module	Global Encoder	CD ↓	F-Score ↑	NC ↑
MARIGOLD	DINO-ViT	16.23	59.97	0.806
MARIGOLD	ViT	17.87 (+1.64)	59.11 (−0.86)	0.789 (−0.017)
MARIGOLD	✗	19.00 (+2.77)	59.24 (−0.73)	0.767 (−0.036)
MiDaS	DINO-ViT	17.48 (+1.25)	59.85 (−0.12)	0.791 (−0.015)
✗	DINO-ViT	21.08 (+4.85)	56.22 (−3.75)	0.778 (−0.028)
✗	✗	24.15 (+7.92)	54.99 (−4.98)	0.770 (−0.036)

## Data Availability

The 3D-FRONT dataset is publicly available at https://tianchi.aliyun.com/specials/promotion/alibaba-3d-scene-dataset (accesed on 15 July 2025), and the Pix3D dataset is available at http://pix3d.csail.mit.edu/ (accesed on 15 July 2025). Code is available here: https://github.com/AnnnnnieZhang/DP-AMF (accessed on 15 July 2025).

## References

[1] Choy C.B., Xu D., Gwak J., Chen K., Savarese S. (2016). 3D-R2N2: A Unified Approach for Single and Multi-view 3D Object Reconstruction. arXiv.

[2] Godard C., Aodha O.M., Brostow G.J. (2017). Unsupervised Monocular Depth Estimation with Left-Right Consistency. arXiv.

[3] Perwez U., Yamaguchi Y., Ma T., Dai Y., Shimoda Y. (2022). Multi-scale GIS-synthetic hybrid approach for the development of commercial building stock energy model. Appl. Energy.

[4] Li Q., Zhao B., Wang X., Yang G., Chang Y., Chen X., Chen B.M. (2025). Autonomous building material stock estimation using 3D modeling and multilayer perceptron. Sustain. Cities Soc..

[5] Palliwal A., Song S., Tan H.T.W., Biljecki F. (2021). 3D city models for urban farming site identification in buildings. Comput. Environ. Urban Syst..

[6] Li Q., Yang G., Bian C., Long L., Wang X., Gao C., Wong C.L., Huang Y., Zhao B., Chen X. (2025). Autonomous design framework for deploying building integrated photovoltaics. Appl. Energy.

[7] Sun L., Jiang Y., Guo Q., Ji L., Xie Y., Qiao Q., Huang G., Xiao K. (2021). A GIS-based multi-criteria decision making method for the potential assessment and suitable sites selection of PV and CSP plants. Resour. Conserv. Recycl..

[8] Li Q., Long L., Li X., Yang G., Bian C., Zhao B., Chen X., Chen B.M. (2025). Life cycle cost analysis of circular photovoltaic façade in dense urban environment using 3D modeling. Renew. Energy.

[9] Wang H., Zhang G., Cao H., Hu K., Wang Q., Deng Y., Gao J., Tang Y. (2025). Geometry-Aware 3D Point Cloud Learning for Precise Cutting-Point Detection in Unstructured Field Environments. J. Field Robot..

[10] Brinatti Vazquez G.D., Lacapmesure A.M., Martínez S., Martínez O.E. (2024). SUPPOSe 3Dge: A Method for Super-Resolved Detection of Surfaces in Volumetric Fluorescence Microscopy. J. Opt. Photonics Res..

[11] Ranftl R., Bochkovskiy A., Koltun V. MiDaS: High-Quality Depth Estimation with Minimal Training Data. Proceedings of the European Conference on Computer Vision (ECCV).

[12] Wang Q., Zhang H., Lin M. Single-view 3D Scene Reconstruction with High-fidelity Shape and Texture. Proceedings of the IEEE Conference on Computer Vision and Pattern Recognition (CVPR).

[13] Oechsle M., Mescheder L., Niemeyer M., Strauss T., Geiger A. Texture Fields: Learning Texture Representations in Function Space. Proceedings of the IEEE International Conference on Computer Vision (ICCV).

[14] Ke B., Obukhov A., Huang S., Metzger N., Daudt R.C., Schindler K. (2024). Repurposing Diffusion-Based Image Generators for Monocular Depth Estimation. arXiv.

[15] Fan H., Su H., Guibas L. A Point Set Generation Network for 3D Object Reconstruction from a Single Image. Proceedings of the IEEE Conference on Computer Vision and Pattern Recognition (CVPR).

[16] Knapitsch A., Park J., Zhou Q.Y., Koltun V. (2017). Tanks and Temples: Benchmarking Large-Scale Scene Reconstruction. ACM Trans. Graph..

[17] Smith J., Wang L., Lee D. Normal Consistency for Surface Reconstruction in 3D Modeling. Proceedings of the European Conference on Computer Vision (ECCV).

[18] Xie H., Yao H., Sun X., Zhou S., Zhang S. Pix2Vox: Context-Aware 3D Reconstruction From Single and Multi-View Images. Proceedings of the 2019 IEEE/CVF International Conference on Computer Vision (ICCV).

[19] Sun B., Jiang P., Kong D., Shen T. (2023). IV-Net: Single-view 3D volume reconstruction by fusing features of image and recovered volume. Vis. Comput..

[20] Groueix T., Fisher M., Kim V.G., Russell B.C., Aubry M. (2018). AtlasNet: A Papier-Mâché Approach to Learning 3D Surface Generation. arXiv.

[21] Shen Q., Yang X., Wang X. (2023). Anything-3D: Towards Single-view Anything Reconstruction in the Wild. arXiv.

[22] Ranftl R., Lasinger K., Hafner D., Schindler K., Koltun V. (2020). Towards Robust Monocular Depth Estimation: Mixing Datasets for Zero-shot Cross-dataset Transfer. IEEE Trans. Pattern Anal. Mach. Intell..

[23] Ranftl R., Bochkovskiy A., Koltun V. (2021). Vision Transformers for Dense Prediction. arXiv.

[24] Kim T., Lee J., Lee K.T., Choe Y. (2024). Single-View 3D Reconstruction Based on Gradient-Applied Weighted Loss. J. Electr. Eng. Technol..

[25] He K., Zhang X., Ren S., Sun J. Deep Residual Learning for Image Recognition. Proceedings of the IEEE Conference on Computer Vision and Pattern Recognition (CVPR).

[26] Dosovitskiy A., Beyer L., Kolesnikov A., Weissenborn D., Zhai X., Unterthiner T., Dehghani M., Minderer M., Heigold G., Gelly S. An Image is Worth 16 × 16 Words: Transformers for Image Recognition at Scale. Proceedings of the International Conference on Learning Representations (ICLR).

[27] Yang W.J., Wu C.C., Yang J.F. (2025). Residual Vision Transformer and Adaptive Fusion Autoencoders for Monocular Depth Estimation. Sensors.

[28] Caron M., Touvron H., Misra I., Jégou H., Mairal J., Bojanowski P., Joulin A. (2021). Emerging Properties in Self-Supervised Vision Transformers. arXiv.

[29] Fu H., Cai B., Gao L., Zhang L.X., Wang J., Li C., Zeng Q., Sun C., Jia R., Zhao B. 3D-FRONT: 3D Furnished Rooms with Layouts and Semantics. Proceedings of the International Conference on Computer Vision (ICCV).

[30] Sun X., Wu J., Zhang X., Zhang Z., Zhang C., Xue T., Tenenbaum J.B., Freeman W.T. Pix3D: Dataset and Methods for Single-Image 3D Shape Modeling. Proceedings of the IEEE Conference on Computer Vision and Pattern Recognition (CVPR).

[31] Liu H., Zheng Y., Chen G., Cui S., Han X. Towards High-Fidelity Single-view Holistic Reconstruction of Indoor Scenes. Proceedings of the European Conference on Computer Vision.

[32] Li J., Wang X., Li D. Total3DUnderstanding: Joint Layout, Object Pose and Mesh Reconstruction for Indoor Scenes from a Single Image. Proceedings of the IEEE Conference on Computer Vision and Pattern Recognition (CVPR).

[33] Xu K., Lin Y., Huang F. (2022). Holistic 3D Scene Understanding from a Single Image with Implicit Representation. IEEE Trans. Pattern Anal. Mach. Intell..

[34] Boulch A., Marlet R. POCO: Point Convolution for Surface Reconstruction. Proceedings of the IEEE/CVF Conference on Computer Vision and Pattern Recognition (CVPR).

[35] Huang J., Gojcic Z., Atzmon M., Litany O., Fidler S., Williams F. Neural Kernel Surface Reconstruction. Proceedings of the IEEE/CVF Conference on Computer Vision and Pattern Recognition.

[36] Li Q., Yang G., Gao C., Huang Y., Zhang J., Huang D., Zhao B., Chen X., Chen B.M. (2024). Single drone-based 3D reconstruction approach to improve public engagement in conservation of heritage buildings: A case of Hakka Tulou. J. Build. Eng..

[37] Liu Y., Chen J. (2023). Research on the Conservation of Historical Buildings Based on Digital 3D Reconstruction. Procedia Comput. Sci..

[38] Shanti Z., Al-Tarazi D. (2023). Virtual Reality Technology in Architectural Theory Learning: An Experiment on the Module of History of Architecture. Sustainability.

[39] Whiton R., Chen J., Johansson T., Tufvesson F. Urban Navigation with LTE using a Large Antenna Array and Machine Learning. Proceedings of the 2022 IEEE 95th Vehicular Technology Conference: (VTC2022-Spring).

[40] Zhang Y., Nakajima T. (2022). Exploring the Design of a Mixed-Reality 3D Minimap to Enhance Pedestrian Satisfaction in Urban Exploratory Navigation. Future Internet.

[41] Long L., Gan Z., Liu Z., Zhao B., Li Q. (2025). MSD-Det: Masonry structures damage detection dataset for preventive conservation of heritage. J. Cult. Herit..

[42] Yang G., Zhao B., Zhang J., Wen J., Li Q., Lei L., Chen X., Chen B. (2025). Det-Recon-Reg: An Intelligent Framework Toward Automated UAV-Based Large-Scale Infrastructure Inspection. IEEE Trans. Instrum. Meas..

[43] Radford A., Kim J.W., Hallacy C., Ramesh A., Goh G., Agarwal S., Sastry G., Askell A., Mishkin P., Clark J. (2021). Learning Transferable Visual Models From Natural Language Supervision. arXiv.

